# Effects of (+)-bicuculline, a GABAa receptor antagonist, on auditory steady state response in free-moving rats

**DOI:** 10.1371/journal.pone.0236363

**Published:** 2020-07-24

**Authors:** Mayako Yamazaki, Sokichi Honda, Keisuke Tamaki, Megumi Irie, Takuma Mihara

**Affiliations:** 1 Department of Neuroscience, Drug Discovery Research, Astellas Pharma Inc., Tsukuba-shi, Ibaraki, Japan; 2 Analysis & Pharmacokinetics Research Labs., Drug Discovery Research, Astellas Pharma Inc., Tsukuba-shi, Ibaraki, Japan; Chiba Daigaku, JAPAN

## Abstract

Auditory steady-state responses (ASSRs) are states in which the electrical activity of the brain reacts steadily to repeated auditory stimuli. They are known to be useful for testing the functional integrity of neural circuits in the cortex, as well as for their capacity to generate synchronous activity in both human and animal models. Furthermore, abnormal gamma oscillations on ASSR are typically observed in patients with schizophrenia (SZ). Changes in neural synchrony may reflect aberrations in cortical gamma-aminobutyric acid (GABA) neurotransmission. However, GABA’s impact and effects related to ASSR are still unclear. Here, we examined the effect of a GABAa receptor antagonist, (+)-bicuculline, on ASSR in free-moving rats. (+)-Bicuculline (1, 2 and 4 mg/kg, sc) markedly and dose-dependently reduced ASSR signals, consistent with current hypotheses. In particular, (+)-bicuculline significantly reduced event-related spectral perturbations (ERSPs) at 2 and 4 mg/kg between 10 and 30 minutes post-dose. Further, bicuculline (2 and 4 mg/kg) significantly and dose-dependently increased baseline gamma power. Furthermore, the occurrence of convulsions was consistent with the drug’s pharmacokinetics. For example, high doses of (+)-bicuculline such as those greater than 880 ng/g in the brain induced convulsion. Additionally, time-dependent changes in ERSP with (+)-bicuculline were observed in accordance with drug concentration. This study partially unraveled the contribution of GABAa receptor signals to the generation of ASSR.

## 1 Introduction

Auditory steady-state response (ASSR) measures the intrinsic ability of auditory neuronal ensembles to entrain to rhythmically presented stimuli and can be used to test the functional integrity of neural circuits that support synchronization [1–3] across frequencies in both human and animal models. Human studies have consistently shown a 40-Hz deficit in ASSR, or in evoked gamma power, in patients with schizophrenia (SZ) [4–8] and in family members with increased risk of SZ [9]. Acute treatment with N-methyl-D-aspartate (NMDA) receptor antagonists like phencyclidine and ketamine is known to mimic a brief SZ-like state in healthy individuals [10–12]. Taking advantage of the clinical evidence, preclinical studies often also employ a short-term disruption to NMDA neurotransmission to mimic a psychosis-like state [13–15]. The effects of NMDA antagonists MK-801 and ketamine have been tested with the 40-Hz ASSR, and results have provided understanding of the role of NMDA receptors in 40-Hz ASSR [16–19]. ASSR is a translatable biomarker which is driving research into neuropsychiatric disorders like schizophrenia.

ASSR deficits in SZ could reflect neurophysiological abnormalities [1, 20] and developmental alterations in the neurotransmission of excitatory (glutamate) and inhibitory (gamma-aminobutyric acid: GABA) transmitter systems [21]. Cortical GABAergic interneurons strongly regulate neuronal network oscillations, particularly in the gamma band [22, 23], and dysfunction of these cells is one of the putative pathophysiological mechanisms of SZ [24, 25].

Furthermore, studies have suggested that ASSR also reflects control by GABA-agonist- activated inhibitory interneurons of the timing of pyramidal neuron firing in cortical layers 2–3 [26], with the interaction between pyramidal neurons and inhibitory neurons thought to underlie the occurrence of neural oscillations [27]. The potential role of the GABA neurotransmitter system in the generation and maintenance of synchronous oscillations [28] has led to increased focus on the system’s possible involvement in the pathophysiology of SZ [29–31], with some neurobiological alterations observed in SZ suggested to result from a compensatory response to restore inhibitory synaptic efficacy [26]. It is likely that the GABAa receptor subtype, which propagates network synchronization [32], is associated with the oscillatory abnormalities observed in patients with SZ. Given that the temporal cortex is thought to play an important role in the generation of ASSRs, these findings suggest a potential link between GABAa abnormalities and ASSR disturbances in SZ. Studies have examined the effects of the GABAa agonist muscimol [33] and GABAa antagonists picrotoxin and (-)-bicuculline methiodide [18, 33] on ASSR in rats; however, the interpretation of these studies has been insufficient and unclear (not significantly).

Here, therefore, we assessed the effects of the GABAa receptor antagonist bicuculline in rats ASSR models which we constructed previously with the same sound protocol in a clinical setting [16]. To evaluate the pharmacological effects of GABAa receptor antagonists, we also examined the pharmacokinetics of bicuculline and its effects on convulsion.

## 2. Experimental procedures

### 2.1 Animals

Sprague-Dawley (SD) rats (Japan Charles River Laboratories International, Inc., Atsugi-shi, Japan) with electroencephalogram (EEG) electrode implants were housed in groups of three in temperature- and humidity-controlled rooms (23 ± 2°C and 55 ± 10%) under a 12-h light/dark cycle. Food and water were available *ad libitum* in the home cages. All animal experimental procedures were approved by the Institutional Animal Care and Use Committee of Astellas Pharma Inc. Tsukuba Research Center is accredited by the Association for Assessment and Accreditation of Laboratory Animal Care International.

### 2.2 Drugs

The GABAa antagonist bicuculline (Sigma-Aldrich Co. LLC., St. Louis, MO, USA) was dissolved in 0.1 mol/L HCl (pH 5.0). The drug was subcutaneously (sc) administered at 1 mL/kg to rats.

### 2.3 Bicuculline concentration in rat brain

Male SD rats aged 11 weeks (Charles River Laboratories Japan, Inc.) that had been freely feeding received a sc dose of (+)-bicuculline (1, 4 mg/kg). The animals were sacrificed under isoflurane anesthesia, and whole blood samples and brain tissue were taken at 10, 30, and 110 min post-dose. Whole blood samples were taken using a syringe containing heparin sodium. After collection of whole blood, the cerebrum was removed from each animal. Whole blood samples were centrifuged to separate plasma. Plasma and cerebrum samples were stored at −20°C until assay.

(+)-Bicuculline concentrations in plasma and 25% brain homogenate samples were determined using a liquid chromatography-tandem mass spectrometry method with a calibration curve range of 3 to 1000 ng/mL. Mean plasma and brain concentrations were calculated using Microsoft Excel ^®^ (Microsoft Corp, Redmond, WA, USA).

### 2.4 Convulsion testing

A convulsion test was performed during the pharmacokinetics testing of bicuculline, using a minimum dose of 1 mg/kg based on Vohs [33] and a maximum dose of 4 mg/kg based on our preliminary results that this dose was not lethal in rats. Regarding efforts to minimize harm and suffering, Astellas Pharma Inc. has acquired AAALAC accreditation and the convulsion experiments were conducted under veterinary supervision. During the convulsion testing, the experimenter observed and monitored the animals from immediately after compound administration until 110 minutes after the end of the experiment. Evaluation was done using a modification of the method of Racine [34]. Convulsion severity was categorized as Grade 0, No convulsion; Grade I, rhythmic mouth and facial twitching; Grade II, rhythmic nodding or tail flicking; Grade III, single limb twitch; Grade IV, bilateral anterior limb rigidity or twitching with standing; and Grade V, comprehensive tonic-clonic with fall. Grade III or higher was judged to indicate induced convulsion. Animals were euthanized after the experiments.

### 2.5 Electrode implantation

Male SD rats aged 8 weeks were anesthetized using isoflurane and then placed into a stereotactic apparatus. The apparatus and experimental procedures were similar to those described previously [16]. After applying lidocaine as a local anesthetic, an incision was made to expose the skull. The location of bregma was identified, and stainless-steel screw electrodes with wire leads were implanted epidurally over the temporal cortex (AP, −4.5 mm; ML, −7.5 mm; and DV, −4.0 mm from bregma), cerebellum (ground), and frontal sinus (reference). Lead wires were connected to the pedestal, and the entire assembly was secured to the skull using dental cement. Recordings were conducted for at least 10 days after the rats recovered from surgery.

### 2.6 EEG recordings

EEG recordings [16] were performed using a programming script with a data acquisition and analysis software package (Spike2^®^, Cambridge Electronic Design, Milton, Cambridge, UK). Rats were hooked to customized electrode cables up to the pedestal. The electrode cables were connected to a high-impedance differential AC amplifier (sampling rate: 1000 Hz, low cut-off filter: 1 Hz, high cut-off filter: 500 Hz; model #1800; A-M Systems, Carlsborg, WA, USA) and versatile data acquisition unit (Micro1401, Cambridge Electronic Design). Rats were individually placed into a recording chamber in an electrically shielded cage with a speaker attached to the top of the cage and moved freely during the EEG recording. For habituation, the EEG recording was started at least 30 min after placement in the recording chamber. Auditory stimuli consisted of click sounds (80 dB, 1 ms), which were presented as 500-ms trains at 40 Hz, with 20 clicks per train. Click sound trains were repeated 200 times/trial, with an inter-train interval of 600 ms. The ASSR was recorded at −10 (baseline), 10, 30, 70 and 110 min after drug injection.

### 2.7 Data analysis

The EEG data from Spike2 were converted for analysis in Matlab. By using the MATLAB toolbox EEGLAB^®^ (MathWorks, Natick MA, USA), signal trial epochs between −250 and 750 ms (for ASSR) or -600 and 0 ms (for baseline gamma power analysis) relative to the first click of the train were extracted from continuous data. All outliers in each split file were rejected for movement artifacts based on a criterion of 2 times the root mean square amplitude per mouse. In ASSR analysis, a low-pass filter (100 Hz) was applied to the EEG data to remove artifacts. Averaging stimuli during each click sound train was performed by wavelet transformation (frequency limits: 10 to 100 Hz, wavelet cycles: 0, epoch size: 1.0 sec). As output data, the measurable factors were divided into two categories: event-related spectral perturbations (ERSPs) (pre-stimulations from −250 to 0 ms) and inter-trial coherence (ITC) using the EEGLAB^®^ and were gathered using open source software, KNIME^®^ (KNIME AG, Zurich, Switzerland). Mean ERSP and ITC were calculated by averaging the data from 100 to 450 msec within a trial (0–500 ms) for 40 Hz (38–42 Hz). ERSP is defined as an event-related change in power relative to a pre-stimulus baseline, while ITC is defined as phase consistency across trials, and ranges between 0 (random phase across trials) and 1 (identical phase across trials) [35]. Baseline power analysis, i.e. stimulus-free and non-time locked analysis, was performed using fast Fourier transform (FFT) analysis with EEGLAB^®^. The value obtained between 30 and 80 Hz was derived from a power spectrum as the baseline gamma power. Both the ASSR and baseline gamma power were expressed relative to pre-dosing values (−10 min). The same animals were used for ASSR and baseline power recordings.

### 2.8 Statistical analysis

For (+)-bicuculline concentration, all values are expressed as mean ± standard deviation (SD). For EEG data, all values are expressed as mean ± standard error of the mean (SEM). Statistical comparisons were performed using one- or two-way analysis of variance (ANOVA) with Dunnett’s multiple comparisons test to compare differences among multiple groups (GraphPad Prism 7^®^, GraphPad Software, San Diego CA, USA). For all tests, *p*<0.05 was considered significant.

## 3. Results

### 3.1 (+)-bicuculline concentration in rat brain (Fig 1)

**Fig 1:**
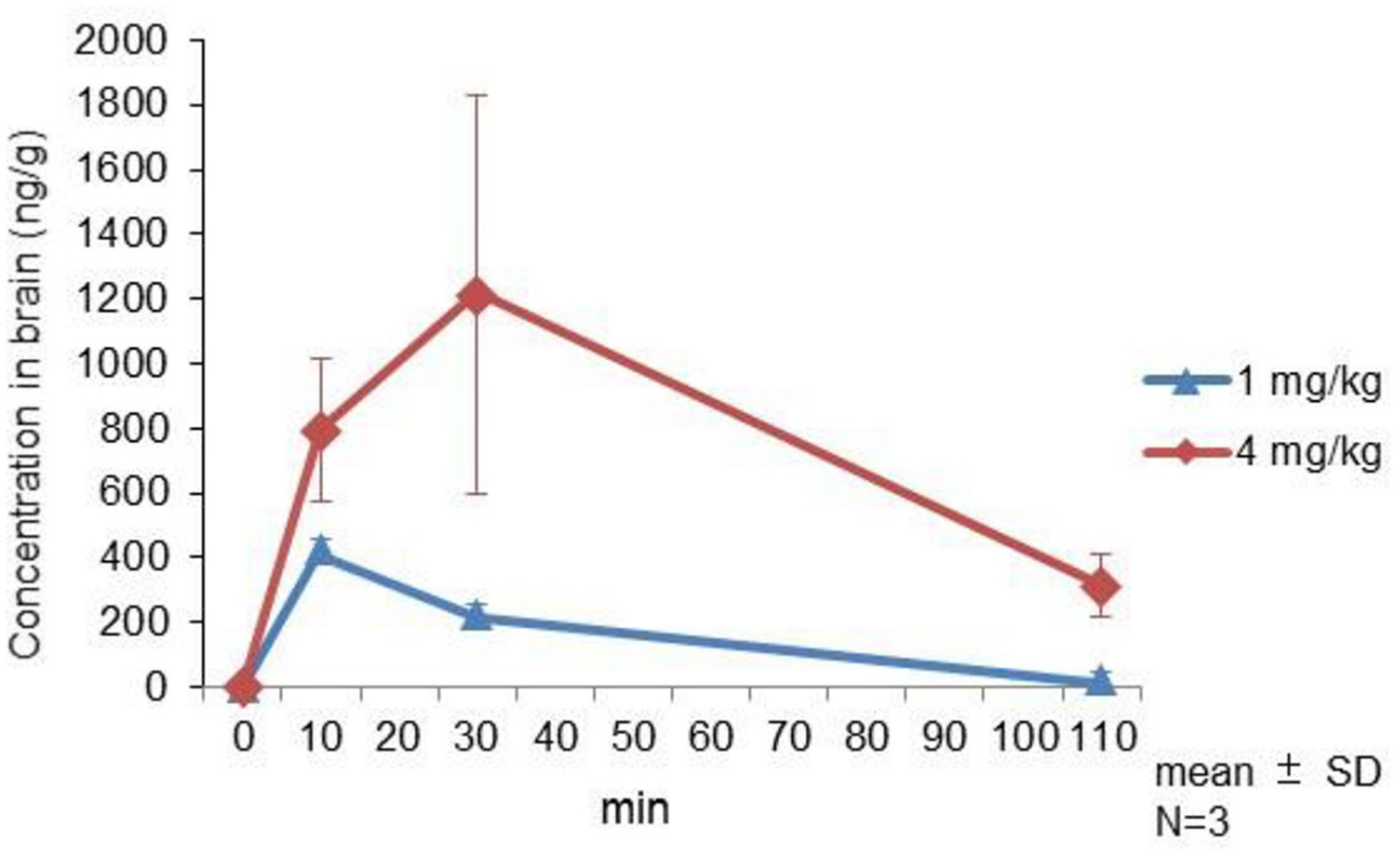
Brain concentrations of (+)-bicuculline in rats. Rats received a subcutaneous dose of (+)-bicuculline (1, 4 mg/kg) in distilled water with 0.1N HCl and 0.1N NaOH (pH 5.0). Animals were sacrificed and the cerebrum was removed at 10, 30, and 110 min post-dose. Brain concentrations were measured by liquid chromatography-tandem mass spectrometry. Values are mean ± SD.

(+)-bicuculline penetrated the brain in a dose-dependent manner and correspondingly induced convulsion at a bicuculline concentration greater than 880 ng/g in the brain. Time to maximum concentration (Tmax) of (+)-bicuculline concentration in the brain was 10 and 30 min at 1 and 4 mg/kg, respectively.

### 3.2 ASSR (Figs 2 and 3)

**Fig 2:**
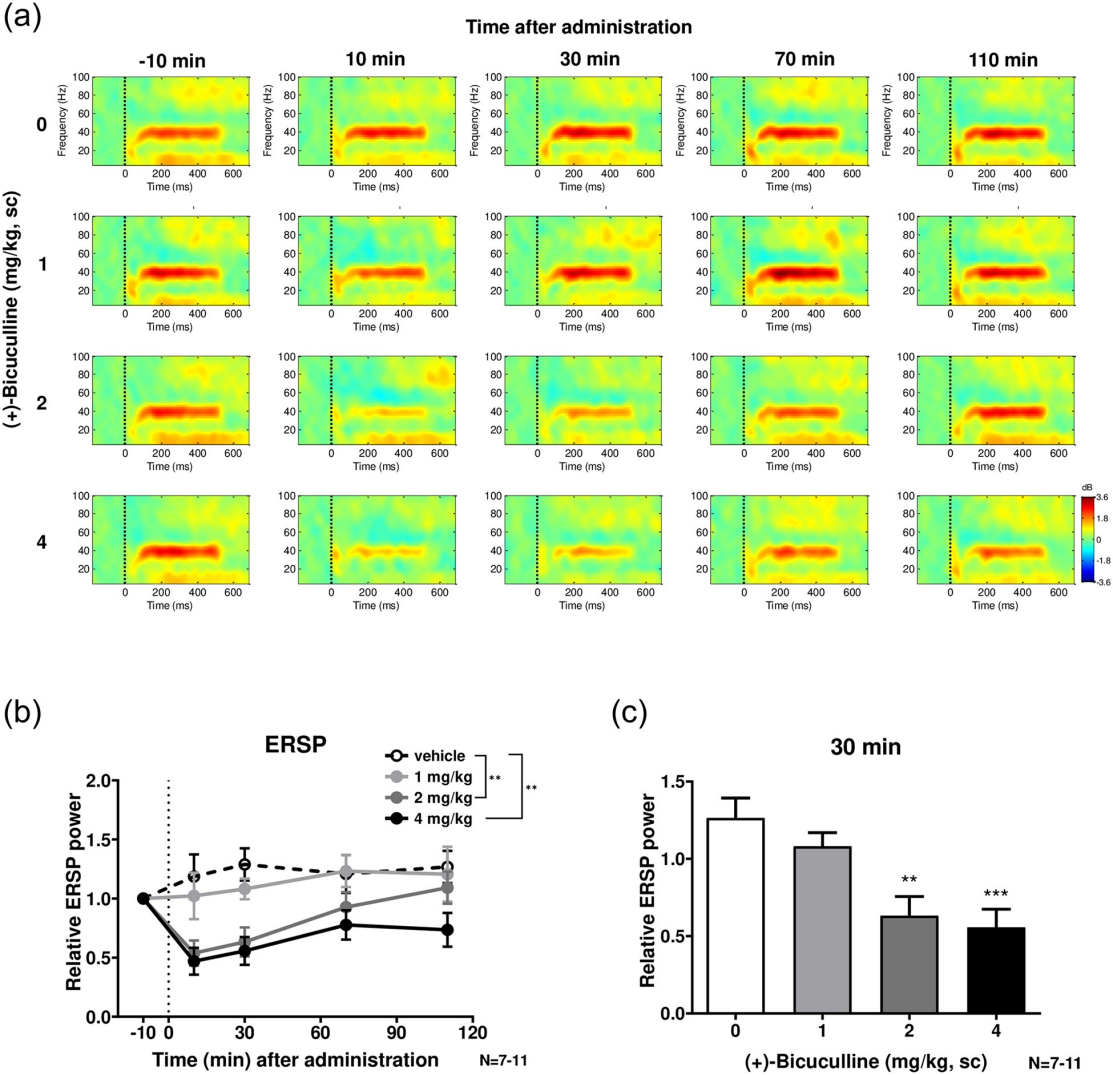
The effect of (+)-bicuculline on the 40 Hz ERSP. **a**, Heat map representation of mean time-frequency plots of ASSR at 40-Hz stimulation at −10 (baseline), 10, 30, 70 and 110 min following (+)-bicuculline (0, 1, 2 and 4 mg/kg, sc) treatment. **b**, Time-course of the 40 Hz ERSP following (+)-bicuculline treatment. **c**, Effect of bicuculline on the 40 Hz ERSP at 30 min after treatment. Bicuculline significantly (4 mg/kg) and dose-dependently decreased ERSP. Values are mean ± SEM. The number of rats was 12 in each group.

**Fig 3:**
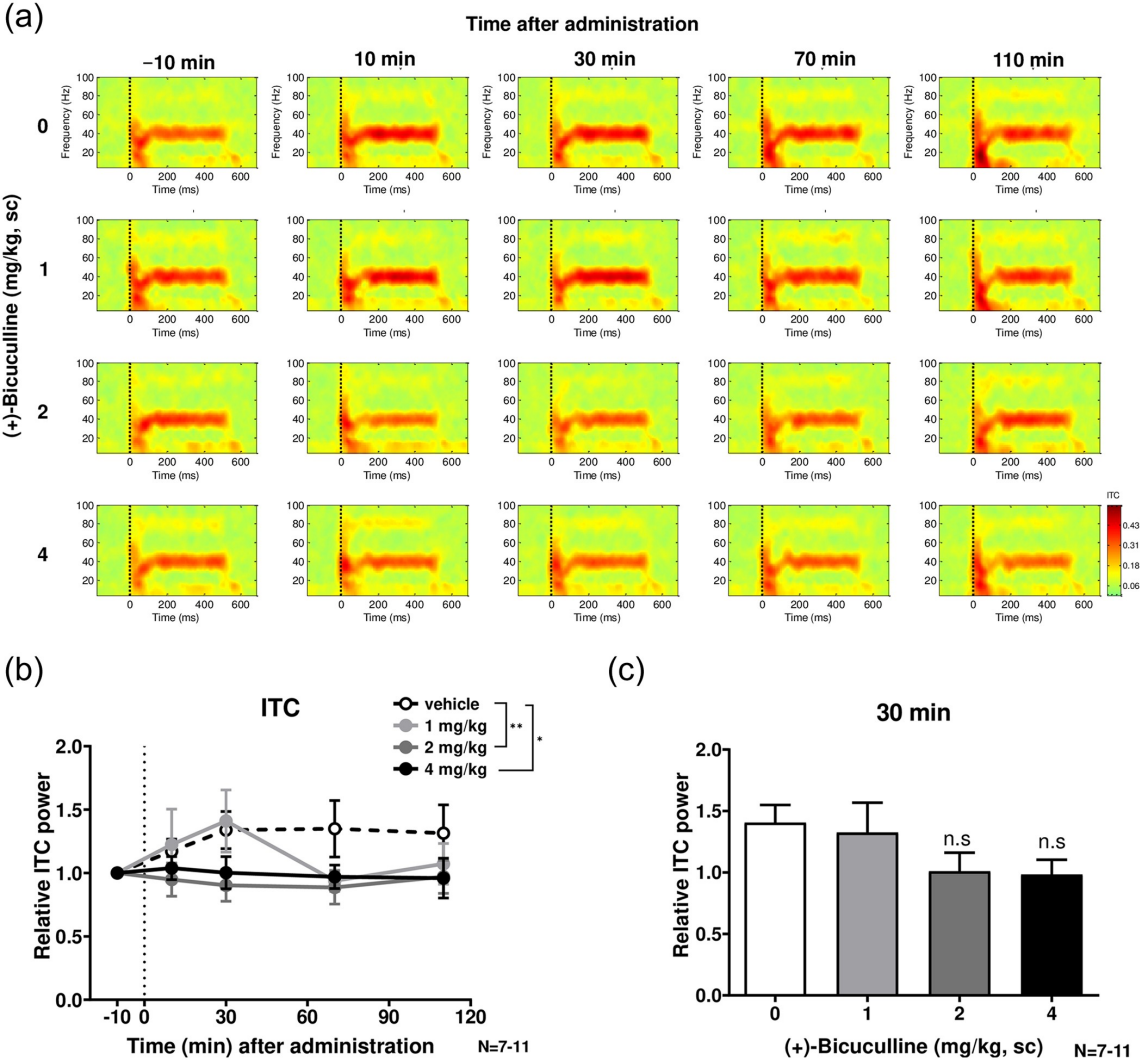
The effect of (+)-bicuculline on the 40 Hz ITC. **a**, Heat map representation of mean time-frequency plots of ASSR at 40-Hz stimulation at −10 (baseline), 10, 30, 70 and 110 min following (+)-bicuculline (0, 1, 2 and 4 mg/kg, sc) treatment, as shown for ERSP in Fig 2. **b**, Time-course of the 40 Hz ITC following (+)-bicuculline treatment. The 40 Hz ITC was significantly reduced in bicuculline (2 and 4 mg/kg)-treated rats than vehicle-treated rats. **c**, Effect of bicuculline on the 40 Hz ITC at 30 min, the time at which bicuculline achieves Cmax. No statistically significant effect was observed on ITC with bicuculline treatment. Values are mean ± SEM. The number of rats was 12 in each group.

Fig 2 shows the effects of (+)-bicuculline on ASSR according to ERSP. Fig 2a shows a heat map representation of mean time-frequency plots of ASSR at 40-Hz stimulation at −10 (baseline), 10, 30, 70 and 110 min following (+)-bicuculline (0, 1, 2 and 4 mg/kg, sc) treatment. The time-course of ERSP of ASSR at 40 Hz following (+)-bicuculline treatment is summarized in Fig 2b. Two-way ANOVA revealed significant time (F(2.274, 75.04) = 4.440; P<0.05) and treatment (F(3, 33) = 8.580; P<0.01) effects. *Post hoc* comparisons showed significantly smaller effects in bicuculline (2 and 4 mg/kg)-treated rats than vehicle-treated rats on Dunnett’s multiple comparisons test (P<0.01). Furthermore, bicuculline significantly (4 mg/kg, p < 0.05, Dunnett’s multiple comparisons test after one-way ANOVA) and dose-dependently decreased ERSP at 40 Hz (Fig 2c) at 30 min, the time at which maximum bicuculline concentration is observed (Cmax; maximum drug concentration).

Fig 3 shows the effects of (+)-bicuculline on ASSR according to ITC at 40 Hz. Fig 3a shows a heat map representation of mean time-frequency plots of ASSR at 40-Hz stimulation at −10 (baseline), 10, 30, 70 and 110 min following (+)-bicuculline (0, 1, 2 and 4 mg/kg, sc) treatment, as was shown for ERSP in Fig 2. In Fig 3b, relative ITC due to treatment at 1, 2 and 4 mg/kg across four time points is compared with that following vehicle treatment. Two-way ANOVA revealed no significant time (F(2.170, 71.61) = 0.6592; P = 0.5323>0.05) or treatment (F(3, 33) = 2.118; P = 0.1167>0.05) effects. However, the ITC of ASSR at 40 Hz was significantly smaller in bicuculline (2 and 4 mg/kg)-treated rats than vehicle-treated rats in Dunnett’s multiple comparisons test. At 30 min, the time at which bicuculline achieves Cmax, the ITC at 40 Hz decreased following treatment with bicuculline (2 and 4 mg/kg, sc), as was observed for ERSP, albeit not significantly so (Fig 3c).

### 3.3. Baseline gamma power (Fig 4)

**Fig 4:**
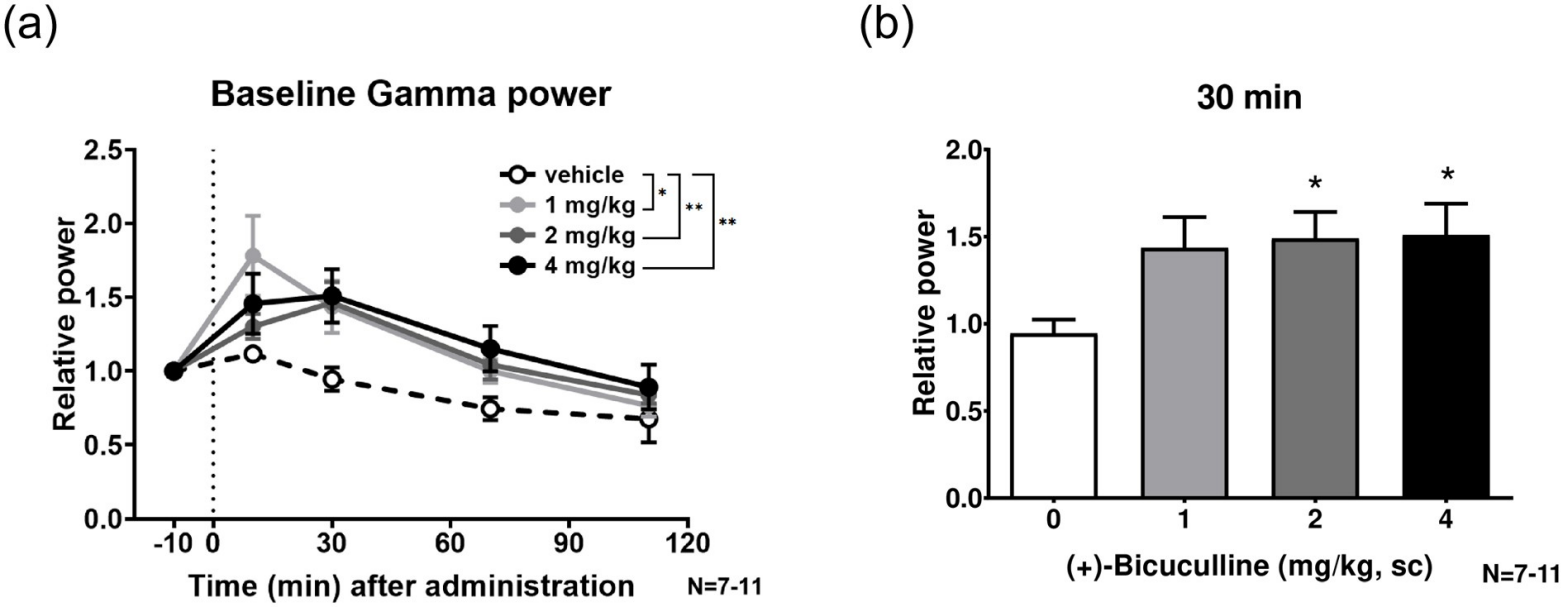
Baseline gamma power (30–80 Hz) following treatment with 1 to 4 mg/kg bicuculline. **a**, Time-course of relative baseline gamma of ASSR following (+)-bicuculline treatment. **b**, Effect of bicuculline on baseline gamma at 30 min. Bicuculline significantly (2 and 4 mg/kg) and dose-dependently increased the baseline gamma power at 30 min.

Fig 4 shows the baseline gamma power (30–80 Hz) following treatment with 1 to 4 mg/kg bicuculline. The time-course of the relative baseline gamma of ASSR following (+)-bicuculline treatment is summarized in Fig 4a. Two-way ANOVA revealed significant time (F(2.297, 75.80) = 21.02; P <0.0001) and treatment (F(3, 33) = 4.200; P<0.05) effects. *Post hoc* comparisons showed a significant increase in bicuculline (1, 2 and 4 mg/kg)-treated rats compared to vehicle-treated rats in Dunnett’s multiple comparisons test (P<0.05, 0.01 and 0.01, respectively). Furthermore, bicuculline significantly (2 and 4 mg/kg, p < 0.05, Dunnett’s multiple comparisons test after one-way ANOVA) and dose-dependently increased the baseline gamma power at 30 min, the time at which the drug achieves Cmax (Fig 4b).

## 4. Discussion

In this study, we examined the effect of the GABAa receptor antagonist (+)-bicuculline on ASSR in free-moving rats. Subcutaneous doses of (+)-bicuculline (1, 2 and 4 mg/kg) caused a marked and dose-dependent decrease the 40 Hz ERSP. In particular, (+)-bicuculline significantly reduced ERSP at 2–4 mg/kg between 10 and 30 min post-dose. (+)-Bicuculline also dose-dependently reduced the 40 Hz ITC, but the efficacy was moderate. Further, baseline gamma power was increased with (+)-bicuculline treatment in a dose-dependent manner. Given that bicuculline is a GABAa receptor-specific full antagonist, disinhibition could always have occurred regardless of the presence or absence of stimulation. ITC is defined as phase consistency across trials and could more directly reflect neural synchrony. In contrast, ERSP is defined as an event-related response relative to prestimulus baseline power, meaning that it would be affected by alteration in baseline gamma power. In the present study, (+)-bicuculline exerted its effects preferentially on ERSP over ITC, suggesting that (+)-bicuculline could preferentially increase baseline gamma power and consequently reduce ASSR.

More importantly, these results for (+)-bicuculline paralleled its pharmacokinetics. Our finding that (+)-bicuculline reduced ERSP suggests that the neuronal activity of pyramidal neurons may be disinhibited regardless of synchronicity because (+)-bicuculline inhibits input signals from GABAergic interneurons to pyramidal neurons. Time-dependent changes in ERSP with (+)-bicuculline occurred in accordance with the drug concentration in brain. Furthermore, the pharmacokinetics of (+)-bicuculline were consistent with its convulsion-inducing effects. Sullivan *et al*. [18] reported that acute and chronic administration of GABAa receptor antagonists had no effect on 40-Hz ASSR despite strong evidence suggesting that GABAergic inhibition is responsible for oscillations and the maintenance of synchronous firing [23, 28, 30]. In that paper, however, the authors used bicuculline methiodide, which may not have penetrated the brain because it has a quaternary amine showing high polarity [36–38]. Drugs that enhance GABAergic signaling have been used to treat seizure disorders since the discovery of phenobarbital in 1912 and the development of benzodiazepines in the 1950s [39]. Soukupová *et al*. [40] and Eder *et al*. [41] reported a focal convulsant action using the hydrophilic form of bicuculline, bicuculline methiodide, on parietal neocortex injection in adult and immature rodents. Graham [42] reported that quaternary bicuculline methiodide (‘N-methyl bicuculline’) [43] and methochloride [44] are much more stable than (+)-bicuculline, and are more water soluble and of similar potency to GABA antagonists, but do not appear to cross the blood brain barrier upon systemic administration. Injected intracisternally, bicuculline methiodide is a more potent convulsant than bicuculline [43]. These studies suggest that bicuculline methiodide does not have the potential to penetrate the brain.

In contrast, (+)-bicuculline penetrated the brain in a dose-dependent manner and induced convulsion at a bicuculline concentration greater than 880 ng/g in the brain. The Tmax of (+)-bicuculline concentration in the brain was 10 and 30 min at 1 and 4 mg/kg, respectively. Johnston *et al*. [44] reported that bicuculline reduces strychnine-insensitive inhibition of pyramidal cells in the cerebral cortex and is a potent convulsant when applied to the cerebral cortex. Taken together, these findings suggest that bicuculline could affect ASSR in the cerebral cortex if sufficient brain concentrations are achieved.

GABA is an important neurotransmitter in the central nervous system. GABAa receptors belong to the family of Cys-loop ligand-gated ion channels [45]. Investigations on the postmortem brains of patients with SZ have revealed abnormalities in GABAergic interneurons, including reduced expression of the GABA-synthesizing enzyme glutamic acid decarboxylase 67 (GAD67) and parvalbumin (PV) in cortical neurons [26, 46, 47]. Furthermore, clinical studies have shown a reduction in GABA in the anterior cingulate cortex as measured using proton magnetic resonance spectroscopy in patients with chronic SZ [48] and first-episode SZ [49]. Nakazawa *et al*. [50] reported that NMDA receptor hypofunction occurs in PV-positive GABA interneurons in early postnatal development, which leads to impairment of cortical maturation, causing a reduction in intrinsic excitability and impaired GABA release and a subsequent disinhibition of pyramidal neurons.

Human studies have consistently shown a 40-Hz deficit in ASSR in patients with SZ [4–8] and in family members with increased risk of SZ [9]. Furthermore, GABAergic (particularly PV-positive) interneurons are disrupted in SZ [51], and the GABAergic system is altered in SZ and PV-positive interneurons offer a potential target for treatment [52]. GABAa receptor expresses in pyramidal neurons and receive input from interneurons [53], suggesting that GABAa receptor hypofunction, such as via administration of a GABAa receptor antagonist, could be a model of SZ. Whether GABAa receptor hypofunction could give rise to the behavioral domain of SZ, i.e. positive symptoms such as hyperactivity and stereotypy, and negative symptoms such as social deficits, as well as cognitive impairment warrants further investigation.

As we mentioned in the Introduction, preclinical studies often employ short-term disruption of NMDA neurotransmission to mimic a psychosis-like state [13–15]. Ketamine is known to alter both the amplitude and latency of auditory ERPs in clinical and preclinical studies [54, 55]. Several groups have used this approach to understand the role of NMDA receptors in the 40-Hz ASSR [16–19], and have demonstrated that NMDA receptor antagonists showed bi-phasic effects; moderate or lower occupancy of NMDA receptors causes augmentation of ASSR while higher occupancy causes blunted ASSR signals. More basically, ketamine causes a persistent reduction in neuronal firing of GABAergic interneurons in the cortex [56, 57], although this would not completely explain the effect of ketamine on ASSR. Note that ketamine is also known to enhance the function of GABAa receptors in cortical neurons [58], which might counteract the hypofunction of interneurons. Blockade of GABAa receptors might therefore be an effective strategy for disrupting ASSR.

In summary, the present study partially unraveled the mechanism of ASSR, namely that GABAa receptors underpin ASSR signal, as has long been hypothesized. This finding helps elucidate the pathogenesis of SZ and will therefore contribute to drug discovery for this condition.

## 5. Conclusion

Subcutaneous doses of (+)-bicuculline markedly and dose-dependently reduced ASSR signals. Furthermore, the occurrence of convulsions was consistent with the drug’s pharmacokinetics. Abnormal gamma oscillations on ASSR are known to be typically observed in patients with SZ. Taken together, this study partially unravels the mechanism of ASSR, and thereby contributes to elucidating the pathogenesis of SZ. ASSR may be a candidate electrophysiological index for GABAergic abnormalities in the auditory cortex.

## Supporting information

S1 Fig Representative image of Event-Related Potential (ERP) during ASSR. For illustration, a representative ERP image (trial by time) from a subject at baseline recording is shown. An averaged ERP wave form is in the panel at bottom. The onset of click trains is set as time zero. A number of epochs is 182 after outlier rejection (see Experimental procedure).
(PPTX)

## Abbreviations

ANOVA: analysis of variance
ASSRs: auditory steady-state responses
EEG: electroencephalogram
ERSPs: event-related spectral perturbations
FFT: fourier transform
GABA: gamma-aminobutyric acid
GAD67: glutamic acid decarboxylase 67
ITC: inter-trial coherence
NMDA: N-methyl-D-aspartate
PV: parvalbumin
sc: subcutaneously
SD: Sprague-Dawley
SD: standard deviation
SEM: standard error of the mean
SZ: schizophrenia
Tmax: Time to maximum concentration
