# Supplementary material for: Effects of (+)-bicuculline, a GABAa receptor antagonist, on auditory steady state response in free-moving rats
Source: PLoS One. 2020 Jul 24;15(7):e0236363. doi: 10.1371/journal.pone.0236363 (PMC7380603; doi:10.1371/journal.pone.0236363)
Supplement: S1 Fig — For illustration, a representative ERP image (trial by time) from a subject at baseline recording is shown. An averaged ERP wave form is in the panel at bottom. The onset of click trains is set as time zero. A number of epochs is 182 after outlier rejection (see Experimental procedure). (PPTX) [file pone.0236363.s001.pptx]

## Slide 1
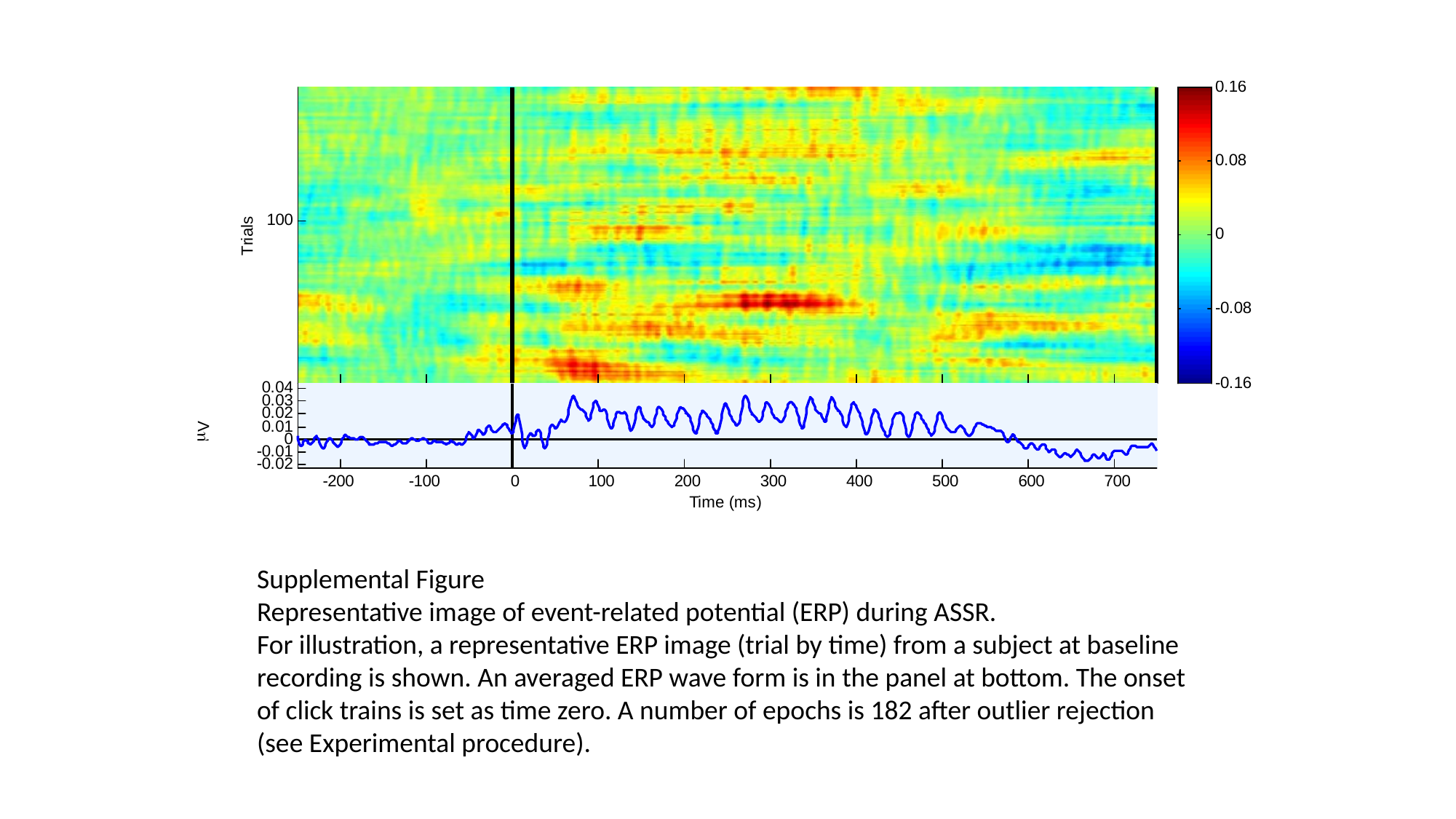

Supplemental Figure
Representative image of event-related potential (ERP) during ASSR.
For illustration, a representative ERP image (trial by time) from a subject at baseline recording is shown. An averaged ERP wave form is in the panel at bottom. The onset of click trains is set as time zero. A number of epochs is 182 after outlier rejection (see Experimental procedure).
